# Cytocompatibility Study of Stainless Steel 316l Against Differentiated SH-SY5Y Cells

**DOI:** 10.3390/biomimetics10030169

**Published:** 2025-03-11

**Authors:** Eleni Zingkou, Asimina Kolianou, Georgios Angelis, Michail Lykouras, Malvina Orkoula, Georgios Pampalakis, Georgia Sotiropoulou

**Affiliations:** Department of Pharmacy, School of Health Sciences, University of Patras, 265 04 Rion-Patras, Greece; asiminakolianou@ac.upatras.gr (A.K.); geangelis@upatras.gr (G.A.); michalislyk@gmail.com (M.L.); malbie@upatras.gr (M.O.); gpampalakis@pharm.auth.gr (G.P.)

**Keywords:** stainless steel 316l, cytocompatibility, differentiated SH-SY5Y cells, neuronal differentiation

## Abstract

Stainless steel (SS) 316l constitutes a popular biomaterial with various applications as implants in cardiovascular and orthopedic surgery, as well as in dentistry. Nevertheless, its cytocompatibility against neuronal cells has not been investigated, a feature that is important for the construction of implants that require contact with neurons, e.g., neuronal electrodes. In addition, most cytocompatibility studies have focused on decorated or surface-modified SS 316l. On the other hand, SH-SY5Y cells are an established cellular model for cytocompatibility studies of potential biomaterials given their ability to differentiate into neuron-like cells. Here, we used retinoic-acid-differentiated SH-SY5Y cells and SH-SY5Y controls to investigate the cytocompatibility and biomimetics of uncoated SS 316l. The assessment of cytocompatibility was based on the determination of differentiation markers by immunofluorescence, RT-qPCR, and the neurite growth of these cells attached on SS 316l and standard tissue culture polystyrene (TCP) surfaces. Even though the neurite length was shorter in differentiated SH-SY5Y cells grown on SS 316l, no other significant changes were found. In conclusion, our results suggest that the uncoated SS 316l mimics a natural bio-surface and allows the adhesion, growth, and differentiation of SH-SY5Y cells. Therefore, this alloy can be directly applied in the emerging field of biomimetics, especially for the development of implants or devices that contact neurons.

## 1. Introduction

Stainless steel (SS) 316l is a chromium–nickel–molybdenum and low-carbon-based alloy with applications in the development of implants applied in cardiovascular surgery, orthopedics, and dentistry. The importance of the chromium addition is based on the generation of a Cr_2_O_3_ passivation layer on the surface that increases its resistance to corrosion. Nickel also protects from galvanic corrosion, while the addition of molybdenum is important from a biomedical point of view, since it generates an alloy that is resistant to pitting corrosion due to chloride ions that are omnipresent in biological fluids [1].

Previous studies have focused on the cytocompatibility testing of SS 316l with osteoblasts and connective tissue cells, as well as hemocompatibility testing [2,3,4]. In this direction, various modifications have also been carried out to increase its cellular affinity and biomimicry, such as tantalum coating [3] or iron-based metallic glass [4]. Here, we assess the cytocompatibility of uncoated SS 316l against neuronal cells. If the uncoated, i.e., non-modified SS 316l is found to be cytocompatible and biomimetic, this will simplify its application in neuronal implants. As a model for neuronal cells, SH-SY5Y neuroblastoma cells were used, which acquire a neuronal-like phenotype upon the induction of differentiation with retinoic acid (RA) [5,6]. Specifically, the neuronal differentiation of SH-SY5Y cells is evident by neurite initiation and guidance. Both processes depend on positive and negative signals from the interaction with the extracellular matrix (ECM) or with other neighboring cells.

Cell adhesion, neurite outgrowth, and differentiation based on molecular markers were compared with cells grown on standard tissue culture polystyrene (TCP) material. In vitro, cell adhesion, proliferation, and differentiation are strongly dependent on the chemical composition of the substrate (matrix). Thus, materials that mimic the natural biogenic surfaces can serve as matrices with important medical applications. The purpose of the current study is to examine the effect of SS 316l as a matrix on the adhesion, proliferation, and differentiation of SH-SY5Y cells. We show that SS 316l can support the growth and differentiation of SH-SY5Y cells, thus potentially expanding its future biomedical applications, e.g., in the generation of neuronal electrodes.

## 2. Materials and Methods

### 2.1. Materials

All chemicals used were obtained from Sigma-Aldrich, St Louis, MO, USA. The SS 316l was purchased from Metall Ehrnsberger GbR (Teublitz, Germany). The plate had a width of 1.2 mm. The SS 316l was cut into 1 cm^2^ and 4 cm^2^ pieces. Tissue culture polystyrene (TCP) was used as a reference material in all experiments. The TCP from the tissue culture plates was also cut into 1 cm^2^ and 4 cm^2^ pieces. All materials were sterilized by exposure to UV for 30 min inside a biosafety class II cabinet. Antibodies were obtained from Cell Signaling Technologies (Danvers, MA, USA) [microtubule-associated protein 2 (MAP2)] and Thermo Scientific (Waltham, MA, USA) (Alexa Fluor 488, donkey anti-rabbit IgG).

### 2.2. Cell Lines

The SH-SY5Y neuroblastoma cells were a gift from Dr. Kostas Vekrellis (Biomedical Research Foundation, Academy of Athens, Athens, Greece) [7].

### 2.3. Cell Culture and Cellular Differentiation

The SH-SY5Y cells were grown in Dulbecco’s Modified Eagle Medium (DMEM, Gibco, Waltham, MA, USA) supplemented with 10% fetal bovine serum (FBS, Gibco, Waltham, MA, USA) and 1% penicillin/streptomycin (Gibco) in a humidified incubator at 37 °C, in an atmosphere of 5% CO_2_. For subculturing, the cells were detached by the addition of trypsin/EDTA (Gibco) followed by incubation at 37 °C for 30–60 s. Thereafter, trypsin was inactivated by the addition of FBS-supplemented medium and the cells were centrifuged at 1000× *g* rpm for 5 min [8,9]. The cell pellet was resuspended in fresh DMEM, and the suspension was aliquoted appropriately in culture flasks for growth. The differentiation of SH-SY5Y cells was induced by the addition of 10 μM RA in the culture medium for 7 days.

### 2.4. Determination of Contact Angle

The contact angle of SS 316l was determined through the sessile drop method with a contact angle goniometer instrument and software (L2004A1, Ossila B.V., Leiden, The Netherlands). Droplets of ultrapure water (density: 0.99 g/mL) were deposited on the materials with the supplied syringe and short videos were recorded. The software allowed for contact angle measurements at different frames when the drop was static. Measurements were analyzed and quantified using OriginPro (2024b).

### 2.5. Culturing SH-SY5Y Cells on SS 316l and TCP Surfaces

Initially, cell viability was verified by the trypan blue exclusion assay (>99%) and the cell number was calculated using a glass hemocytometer (Neubauer, Marienfeld Superior, Lauda-Königshofen, Germany). Sterile SS 316l and TCP pieces were placed in tissue culture 6-well plates. In total, 60,000 SH-SY5Y cells suspended in DMEM were deposited on 4 cm^2^ material pieces. The cells were allowed to adhere for 6 h, then fresh medium (3 mL) was added to completely cover the SS 316l and the TCP surface. On the following day, fresh medium containing 10 μM RA was added to the wells for cell growth and differentiation [5]. The materials were removed for analysis at the designated time points.

### 2.6. Morphological Assessment of Differentiation Status

SH-SY5Y cells growing on TCP were monitored on an inverted phase-contrast microscope and the cells grown on SS 316l were observed by immunocytochemistry and scanning electron microscopy (SEM). Following differentiation, axon length (neurite outgrowth) was measured using the open-source ImageJ software (1.54g) and the distribution curves were plotted using OriginPro (2024b).

### 2.7. Scanning Electron Microscopy (SEM) 

The surface of the SS 316l was inspected without gold sputtering on a field emission scanning electron microscope (FE-SEM, JEOL, 6300, JEOL, Tokyo, Japan) coupled with an energy dispersive X-ray spectrometer (EDX) for visual and elemental analysis. SH-SY5Y cells cultured on SS 316l were fixed in 4% paraformaldehyde (PFA) in phosphate-buffered saline (PBS) at pH 7.2 for 20 min and dehydrated in a series of 5 min incubations in solution with increasing ethanol concentrations (50% to 100%). Finally, the samples were air-dried, gold sputtered (10 nm layer), and imaged in the same FE-SEM. To this end, it is noted that the uncoated SS 316l surface was inspected directly with SEM, while, when cells were attached on the surface, the sample was gold sputtered first, then inspected with SEM.

### 2.8. Immunofluorescence

Differentiated SH-SY5Y cells cultured either on TCP or on SS 316l were initially fixed by incubation with 4% PFA in PBS at pH 7.2 for 20 min and washed with 0.05% Tween-20 in PBS. The cell membranes were permeabilized by incubation in 0.1% Triton-X-100 in PBS for 5 min. Subsequently, the cells were rinsed with 0.05% Tween-20 in PBS and submerged in blocking solution [1% bovine serum albumin (BSA) in PBS] for 30 min and incubated overnight at 4 °C with an antibody specific for the MAP2 protein (MAP2, 1:400, Cell Signaling Technologies, Danvers, MA, USA) [10]. On the following day, the cells were incubated for 30–60 min with a secondary antibody (Alexa Fluor 488, donkey anti-rabbit IgG, 1:1000, Thermo Scientific, Waltham, MA, USA) emitting at 520 nm. Actin filaments were mapped with tetramethylrhodamine (TRITC)–phalloidin (1:500) using the actin cytoskeleton and Focal Adhesion Staining Kit (FAK100 Kit, Millipore, Burlington, MA, USA) [11]. Finally, the nuclei were counterstained with 4′,6-diamidino-2-phenylindole (DAPI, 1:1000) and the materials were mounted on microscope slides using an antifade mounting solution (Mowiol, Sigma-Aldrich, St Louis, MO, USA) [12] before visualization with a fluorescence microscope (Axioscope 5, Carl-Zeiss, Oberkochen, Germany) coupled with a Zeiss Axiocam 208.

### 2.9. Reverse Transcription Quantitative PCR (RT-qPCR)

The total RNAs were extracted from the differentiated and undifferentiated SH-SY5Y cells using Nucleospin by Macherey-Nagel. For real-time (quantitative) RT-PCR, the SuperScript First-Strand Synthesis System (Invitrogen, Waltham, MA, USA) was used, and the resulting cDNAs (50 ng) were amplified with SYBR and gene-specific primers for the genes encoding microtubule-associated protein 2 (*MAP2*) and neurofilament heavy protein subunit (*NEFH)*. Gene expression was normalized against the gene encoding for hypoxanthine phosphoribosyltransferase 1 (*HPRT-1*). The gene-specific primer sequences are given in Table 1.

### 2.10. Cellular Proliferation

The cells were detached with trypsin, as mentioned. The pellet was resuspended in fresh medium. A small fraction of the cell suspension was mixed with 0.4% trypan blue dye at a 1:1 ratio and inserted into a glass hemocytometer chamber. The healthy cells possessed intact cell membranes which were impervious to trypan blue, while dead cells’ membranes were compromised, thus allowing the dye to penetrate and stain the cell. Cell viability was estimated using the following equation: [number of living cells/(number of living cells + number of dead cells)] * 100. The cell density (cells/mL) was calculated according to the following formula: [total number of counted cells/(number of counted squares * volume of 1 square)] * (dilution factor). The volume of one hemocytometer square equaled 0.1 mm^3^. Total protein was quantified in the SH-SY5Y cell lysates using the Bradford reagent (Sigma-Aldrich). Lysis of cells was carried out with the radioimmunoprecipitation assay (RIPA) buffer [25 mM Tris-HCl, pH 7.6, 150 mM NaCl, 1% NP-40, 1% sodium deoxycholate and 0.1% sodium dodecyl sulfate (SDS)]. The absorbance at 595 nm was recorded and the protein concentration was determined by comparison to a standard curve generated with BSA.

### 2.11. Determination of Leaching Elements

SS 316l was submerged for 31 days in PBS and incubated at 37 °C. This buffer mimics physiologic conditions and was selected to emulate a natural biocorrosion process. The material was subsequently examined by SEM/EDX. The recovered PBS was analyzed by X-ray fluorescence (XRF) (S2 Picofox, Bruker, Billerica, MA, USA) to determine the presence of metals leaching from SS 316l.

For XRF analysis, Sample A was prepared by mixing 495 μL of the initial sample with 5 μL of gallium (Gallium High-Purity Standards 1000 mg/L, Charleston, SC, USA), which was added as an internal standard (concentration of 10 mg/L). Subsequently, 450 μL of the initial sample was mixed with 50 μL of Sample A, so that a Sample B was prepared with a final concentration of Ga with an internal standard equal to 1 mg/L. The vortex of each Sample B for 10 s followed. A total of 10 μL of each Sample B was placed on the quartz sample holder and dried using a hot plate at 50 °C for 10 min. The parameters for XRF were as follows: excitation, Mo K; voltage, 50 kV; current, 600 μA; live time, 1000 s; and sample holder, quartz glass sample carrier.

### 2.12. Collagen Coating and microRAMAN Spectroscopy (RAMAN Microscopy)

Collagen coating was performed with rat-tail collagen type I (Corning, Glendale, AZ, USA), which is the most widely used collagen for cell culture experiments [13]. The rat-tail collagen type I solution was diluted to 2.5 mg/mL with sterile water, the solution was deposited on the surface of SS 316l (250 μg/cm^2^), and the coated piece was incubated at 4 °C overnight. On the following day, after discarding any excess collagen solution, the piece was further incubated at 37 °C for 24 h. The coated SS 316l piece was, finally, rinsed with PBS and sterile water to remove unadhered protein and then air-dried. The binding of collagen on the surface was determined with microRAMAN spectroscopy (LabRam HR Evolution, Horiba, Kyoto, Japan). RAMAN datapoints were normalized against amide I peaks [14] and smoothened with the Savitsky–Golay method for improved visual clarity [15].

### 2.13. Indirect Cytocompatibility

The material extracts were prepared in accordance with part 12 “Sample preparation and reference materials” of ISO 10993 [16]. (https://www.iso.org/standard/75769.html, accessed on 27 October 2024). Specifically, the 4 cm^2^ SS 316l and the TCP materials were incubated in 3 mL serum-free culture medium for 24 h. The extracts were collected and supplemented with 10% FBS before administering them to cells. SH-SY5Y cells were seeded in 96-well plates at a density of 10,000 cells per well, then incubated at 37 °C, 5% CO_2_, for 24 h. Following this, the cells were differentiated with RA for 7 days. On the next day, the cells were exposed to the tested extract for 24 h and examined under a phase contrast microscope (Carl Zeiss) to identify the growth characteristics of control and treated cells. Finally, cytotoxicity was quantified by the 3-(4,5-Dimethylthiazol-2-yl)-2,5-diphenyltetrazolium bromide (MTT) assay and neutral red uptake (NRU) assay according to part 5 “Tests for in vitro cytotoxicity” of ISO 10993 (https://www.iso.org/standard/36406.html, accessed on 27 October 2024).

For the MTT assay, a solution of MTT in sterile PBS was prepared fresh (5 mg/mL). The MTT solution was then combined with an equal volume of serum-free DMEM and applied to the cells (100 μL/well). The cells were incubated at 37 °C until blue formazan crystals were visible under the microscope. The MTT medium was removed and 100 μL dimethyl sulfoxide (DMSO) was added in each well to dissolve the formazan crystals. The absorbance was measured in a plate reader at 570 nm. For each experiment, blanks, untreated cells, and cells treated with 1% SDS were included as controls.

For the NRU assay, one day prior to testing, neutral red (NR) solution 0.4% was added to the culture medium (1:80) and incubated at 37 °C overnight. The following day, the NR medium was filtered to discard NR crystals that may have formed. The culture medium was then removed, the cells were washed with PBS, and the NR medium was added to each well, including the blanks (100 μL/well). The plates were incubated at 37 °C for 1–3 h, then the NR medium was discarded. The cells were washed with PBS (150 μL) and a destaining solution (50% ethanol, 1% acetic acid) was added. The absorbance was recorded with a plate reader at 540 nm.

### 2.14. Statistical Analysis

All data are expressed as mean ± standard deviation (SD) or standard error of the mean (SEM), as indicated in the figure legends. Statistical analyses were performed using OriginPro (2024b) and GraphPad (10.4). The differences between groups were assessed using an unpaired t-test. A *p*-value of less than 0.05 was considered statistically significant.

## 3. Results

### 3.1. Morphology, Elemental Analysis, and Potential Corrosion of Stainless Steel 316l

**Figure 1:**
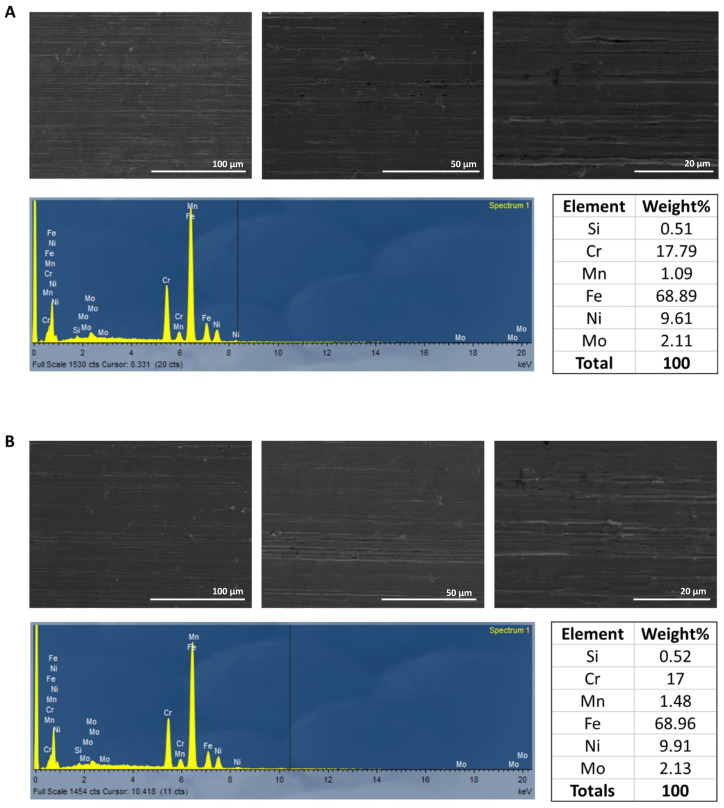
Surface characterization of SS 316l before (**A**) and after immersion in PBS for 31 days (**B**). It is easily observed that no alteration of SS 316l composition or structure occurred during this period, indicating that this material is indeed resistant in solutions containing ions found in physiological fluids (mainly Cl^−^).

Initially, SEM and EDX analysis were conducted on a sample of SS 316l to verify its elemental composition. As shown in Figure 1A, the SS 316l contained iron, chromium, nickel, and molybdenum, as expected [1]. Further, no major defects on its surface were detected. Then, a sample of SS 316l was incubated in PBS for 31 days to investigate whether corrosion of the alloy would take place. PBS was selected since it mimics the osmotic pressure and the ion concentrations found in the human body, especially the concentrations of Cl^−^ [17]. As shown in Figure 1B, no morphological alterations on the surface of SS 316l were detected and the elemental composition was identical to the sample before being exposed to PBS. To further investigate whether the alteration of SS 316l had taken place, the PBS solution where the sample was immersed for 31 days was withdrawn and analyzed for wear metals (Fe, Cr, Ni, Mn) with XRF. As shown in Table 2, wear metals were detected in a very low concentration, indicating that corrosion was most likely limited. Mo was undetectable with XRF.

### 3.2. Contact Angle Measurement

**Figure 2:**
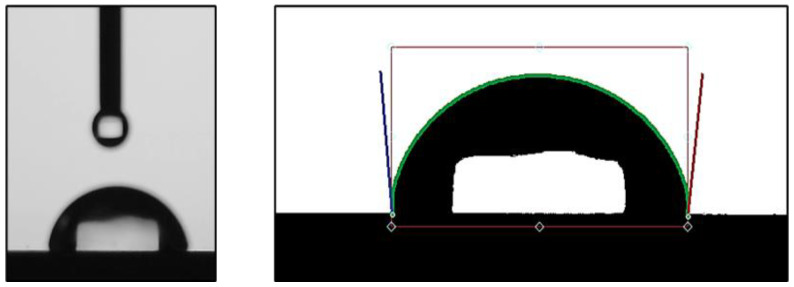
Contact angle measurement. **Left**: actual image of water drop on the surface. **Right**: processed image and analysis with Ossila’s software (version 4.2.1). Blue color indicates the contact angle measurement on the left and dark red on the right side of the droplet.

Contact angle is a measure of the hydrophilicity/hydrophobicity of a surface. Usually, surfaces that are highly hydrophobic cannot support cellular growth [18]. The contact angle of the SS 316l was found to be 94.73° ± 8.07° (Figure 2). The relatively large SD is probably related to the fact that the surface of SS 316l is not “ideal”. Instead, it displays many micro-irregularities in its microstructure (Figure 1) that directly affect the contact angle.

### 3.3. Cellular Adhesion and Proliferation on SS 316l Surface

**Figure 3:**
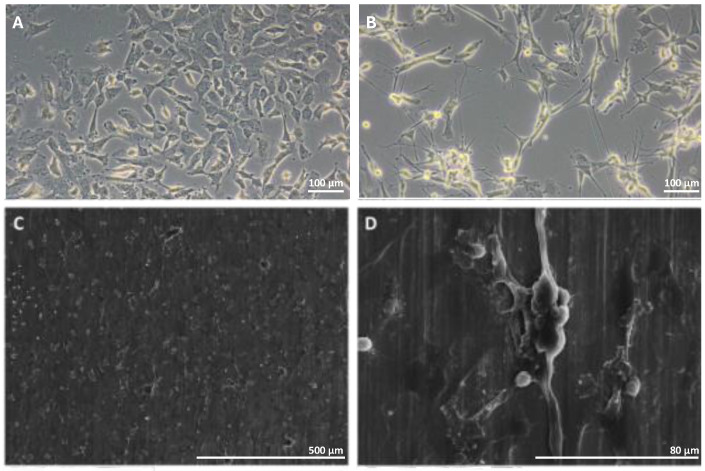
Morphological appearance of SH-SY5Y cells undifferentiated (**A**) and differentiated on TCP (**B**) and SS 316l (**C**,**D**). Undifferentiated SH-SY5Y cells appear small and oblong without long axial projections while differentiated SH-SY5Y cells appear more “neuron-like” and create a neurite network.

The cells were deposited on the surface of SS 316l as described in Materials and Methods and treated with RA for 7 days. On day 7, the morphology of the cells was tested with optical microscopy for cells grown on TCP and SEM for cells grown on SS 316l (Figure 3B, Figure 3C, and Figure 3D, respectively). As shown in Figure 3, SH-SY5Y cells exposed to RA changed their morphology to more elongated cells with neurite outgrowths, indicating that cells were subjected to differentiation in both substrates, at least at the micromorphological level.

**Figure 4:**
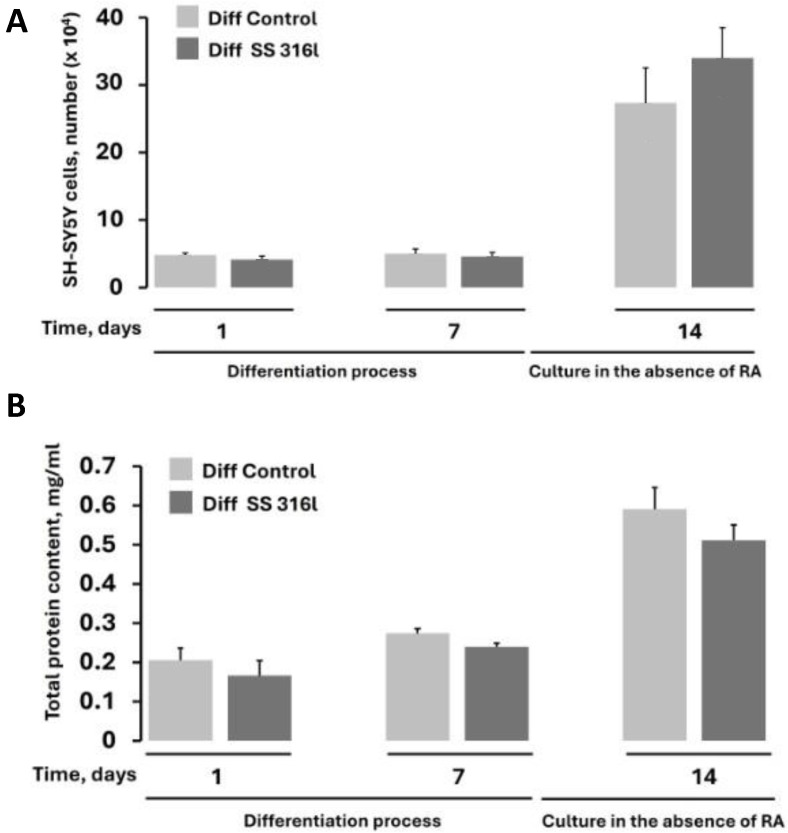
Total cell number (**A**) and protein content (**B**) of SH-SY5Y cells deposited and differentiated on control material (TCP) or SS 316l. Values are shown as ±SD. No statistically significant difference was found (*p* > 0.05).

Cellular adhesion was determined by calculating the number of cells that were attached on the TCP and SS 316l 24 h (day 1) after spotting the cellular suspension. As shown in Figure 4A, there were no statistically significant differences between the TCP and SS 316l; therefore, the surfaces can both support cellular attachment to the same extent. We also determined the total protein at d1, which directly relates to the number of cells. As shown in Figure 4B at d1, no statistically significant differences were found between the TCP and SS 316l, which is consistent with the result shown in Figure 4A. Then, the number of cells on d1 and d7 were compared. As shown in Figure 4, the number of cells between d1 and d7 is identical. This is consistent with the fact that within this period, the cells were induced to differentiate with the addition of RA, and therefore, they did not proliferate. In addition, after d7, the cells were further incubated for 7 days in the absence of RA but in the presence of serum (d14), which allowed for cellular proliferation. The number of cells and the total protein content verified the induction of proliferation from d7 to d14 (Figure 4). Collectively, SS 316l does not affect the attachment and growth of cells and has potential biomimetic properties.

### 3.4. Molecular Characterization of SH-SY5Y Cell Differentiation on SS 316l Surface

**Figure 5:**
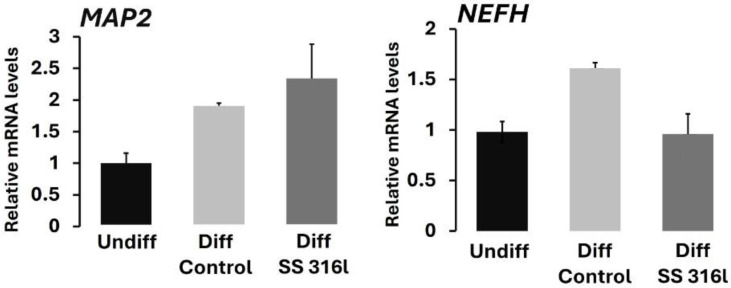
RT-qPCR analysis of *MAP2* and *NEFH* expression. The values are shown as ±SD.

To better characterize the differentiation process of SH-SY5Y cells on SS 316l, the expression of *NEFH* (encoding for neurofilament heavy chain) and *MAP2* (encoding for microtubule-associated protein 2) were determined by RT-qPCR for cells exposed to RA for 7 days. As shown in Figure 5, the upregulation of *MAP2* was detected, while *NEFH* was only upregulated in SH-SY5Y cells grown on TCP.

**Figure 6:**
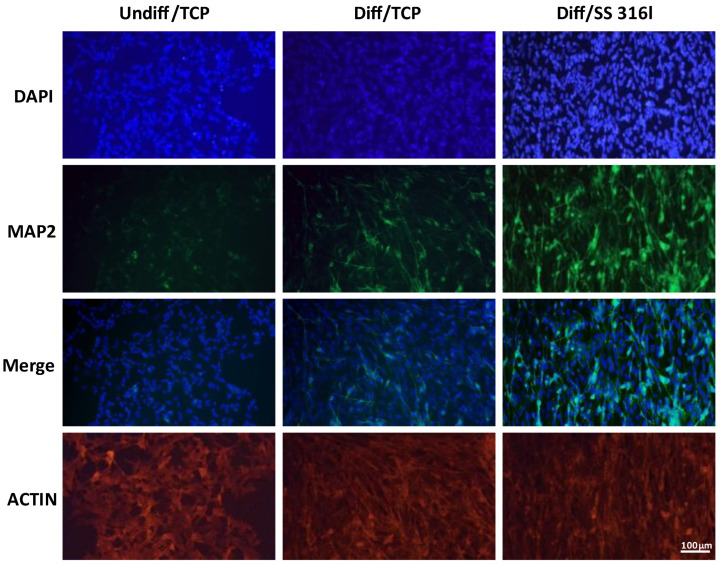
Analysis of MAP2 expression and the structure of the actin cytoskeleton with immunofluorescence. Nuclei were stained blue with DAPI. Merge indicates the merging of DAPI with MAP2. Actin was stained with phalloidin. The images were captured at 10× magnification with a fluorescence microscope. Scale bar: 100 μm.

To this end, it should be noted that the most important marker for the differentiation of SH-SY5Y cells is MAP2 [19], and this marker has been used to study the differentiation status of SH-SY5Y cells grown on various surfaces [20]. Thus, the expression of MAP2 at the protein level was also tested with immunofluorescence. In addition, phalloidin staining was conducted to visualize the F-actin filaments in differentiated SH-SY5Y cells (Figure 6).

**Figure 7:**
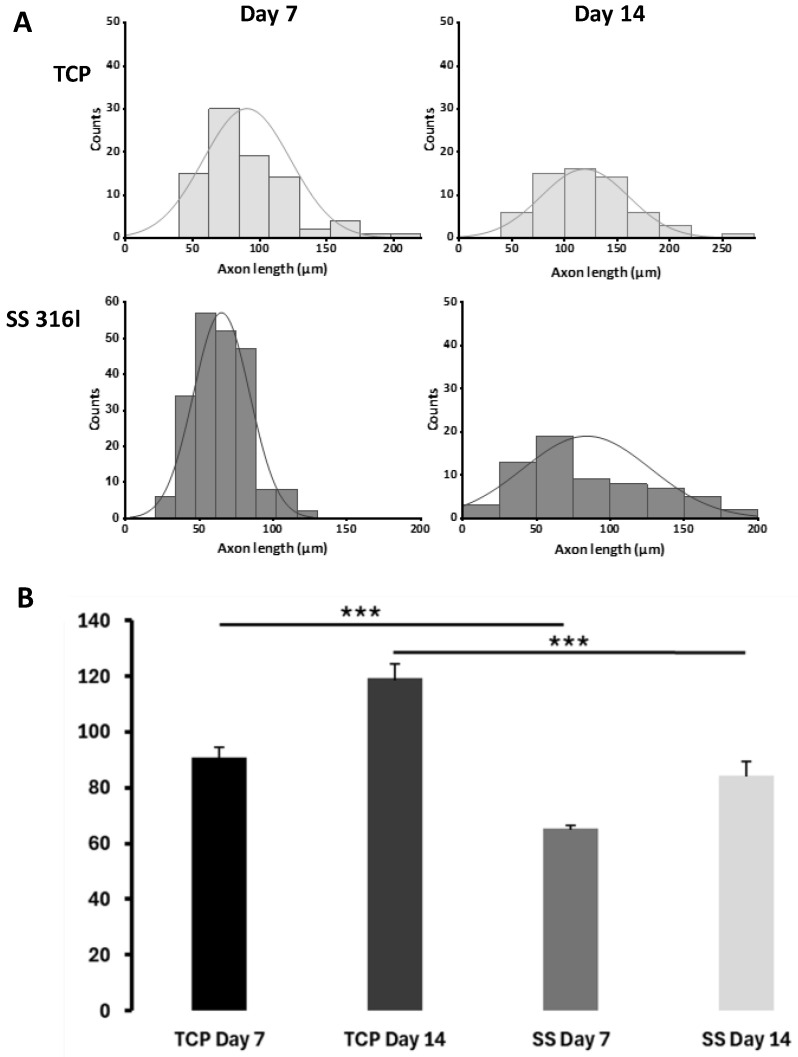
Distribution curves (**A**) and quantification of the mean axonal length (**B**) of differentiated SH-SY5Y cells grown on the TCP and SS 316l surfaces for 7 days and 14 days. The data are presented as ± SEM. The axon length value difference among SS 316l and the corresponding TCP control measured on days 7 and 14 is statistically significant (***: *p* < 0.0001).

Based on the immunofluorescence staining for MAP2, the length of the developed neurites was measured and quantified. As shown in Figure 7, SH-SY5Y cells grown on TCP exhibit longer axon lengths compared to cells grown on SS 316l both on d7 and on d14.

### 3.5. SS 316l Supports Collagen Coating

**Figure 8:**
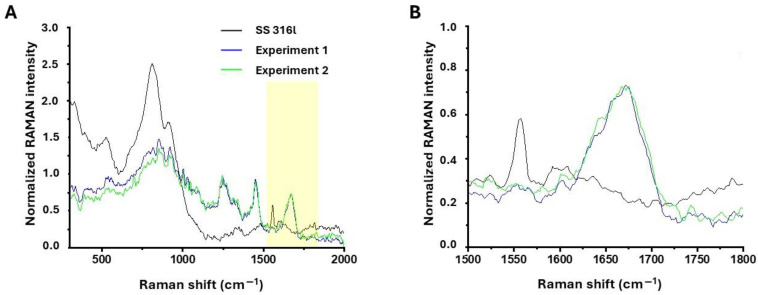
Surface analysis of uncoated and coated SS 316l with RAMAN microscopy. (**A**) RAMAN spectra from the surface of uncoated SS 316l and SS 316l coated with collagen I. The experiment was carried out in duplicate (Experiment 1 and 2). (**B**) The yellow region in (**A**) is magnified to show the amide I region (1600–1700 cm^−1^). SS 316l does not show any peak in this region that may interfere with the amide I. Therefore, it is easily concluded that collagen has decorated the SS 316l surface.

To demonstrate that the surface of SS 316l can be directly coated by the ECM that is required to support the growth of cells, we incubated the SS 316l with rat collagen type I solution and then studied the adsorption of collagen with RAMAN microscopy. As shown in Figure 8, the detection of the characteristic collagen amide I band [21,22] occurs only in the samples with deposited collagen. This indicates that collagen I could bind on the surface of SS 316l. We have also analyzed the SS 316l surface with SEM after the deposition of collagen I and we have not detected any difference from the uncoated surface. This is logical, since the adsorption of collagen is expected to yield a very thin layer that is only detected with RAMAN spectroscopy.

### 3.6. Assessment of Cytocompatibility of SS 316l with Indirect Assay

**Figure 9:**
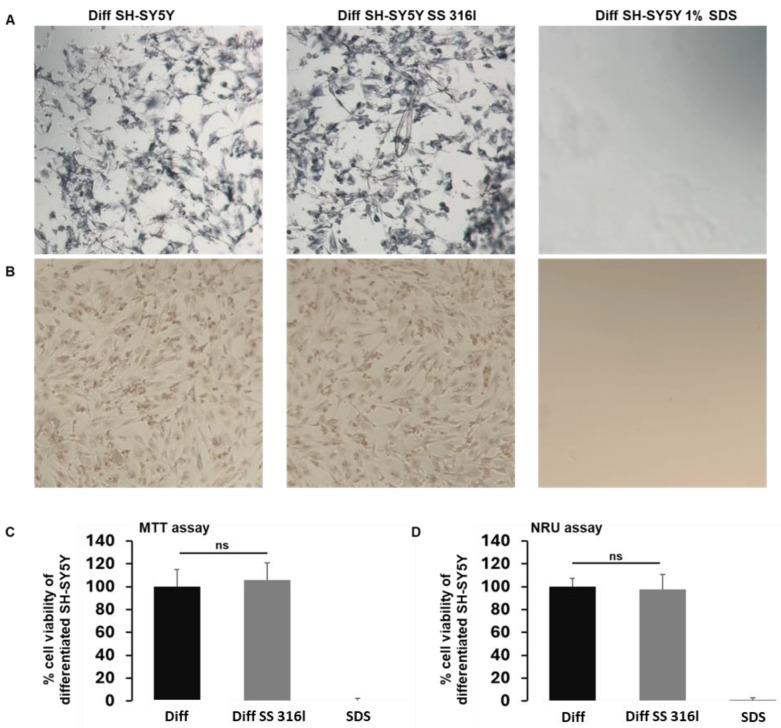
Assessment of in vitro cytotoxicity of SS 316l extract on differentiated SH-SY5Y cells. (**A**) Representative microphotographs of differentiated SH-SY5Y cells incubated with the SS 316l extract for 24 h after the application of MTT. The formazan crystals have stained the cells. (**B**) Staining of SH-SY5Y cells with neutral red (NR). Quantification of cellular viability with MTT (**C**) and neutral red (**D**). Data are expressed as % viability of differentiated cells and are presented as median ± SD from two independent experiments. All reactions were set up in triplicate in each experiment and 1% SDS was applied as a control since it lysed the cells. ns: not significant.

Further, we performed an indirect cytocompatibility assay based on the ISO 10993 guidelines. These guidelines require the preparation of a material extract for 24 h. As shown in Figure 9 and in accordance with our previous data, no cytotoxicity was observed in the SH-SY5Y cells treated with SS 316l extract.

## 4. Discussion

SH-SY5Y cells represent a classical model to study differentiation, as well as to study the effect of various compounds on neurite outgrowth [23]. In previous studies, various substrates were used as biomimetic scaffolds to support the differentiation of SH-SY5Y cells, including surfaces with 3D microarchitecture generated by the deposition of carbon nanotubes on glass [20]. Our data suggest that uncoated SS 316l can support the viability, growth, and differentiation of SH-SY5Y cells. Further, no signs of cytotoxicity were observed, since the morphology of the cells was normal, cellular proliferation was normal, and the number of cells adhered on the surface of SS 316l was indistinguishable from the TCP.

Recently, the coating of SS 316l with a sublayer of titanium or chromium and then with diamond-like carbon was suggested to increase the corrosion resistance for biomedical implants [24]. Nevertheless, its cytocompatibility and its ability to support neuronal growth were not tested. Here, corrosion resistance was demonstrated by incubating the SS 316l in PBS. The corrosion of implants within the human body will not only compromise the functionality of the device but could endanger the health of the individual. Nonetheless, we found a very small amount of wear during incubation with PBS. The absence of cytotoxicity was confirmed by two different assays: the trypan blue dye exclusion assay and the measurement of total protein as a marker for the cell number. Both assays were in accordance with expectations. The reason for using these assays instead of the MTT assay was based on the fact that the SS 316l substrate is opaque. Therefore, the reduction of MTT by cells cannot be followed with microscopy to identify the time point to stop the reaction.

To this end, it should be mentioned that protein coating reduces the wear of the metal surfaces, as was demonstrated with the application of BSA on the surface of SS 316l [25]. It is noteworthy that, to attach to a surface, the cells first secrete proteins of ECM that, in turn, are adsorbed on the surface, creating an ECM-like scaffold on which the cells adhere. This scaffold provides better protection than the uncoated SS 316l used in the PBS corrosion experiment.

Also, here we did not find a negative effect on the differentiation of SH-SY5Y cells exerted by the SS 316l, except for a shorter neurite length. The main biochemical marker of differentiation (MAP2) was identical between the cells grown and differentiated on SS 316l and TCP. As expected, during the differentiation process, the number of SH-SY5Y cells remained almost constant (d1 and d7, Figure 4) as determined by both trypan blue assay and total protein content. However, upon the removal of RA and the addition of serum, the cells were allowed to proliferate as expected, and on day 14 their number had increased considerably (d14, Figure 4). The shorter neurite outgrowth may indicate that a protein coat may be applied on the surface of SS 316l to improve its biomimetic performance. Indeed, it has been reported that the attachment of cells on protein surfaces increases the neurite outgrowth and increases the repeatability of neurite outgrowth behavior [26]. Nevertheless, for in vivo applications, this may not be very important, since the implants will be in contact with neurons that are fully differentiated within an organism.

To support our findings, we have also performed an indirect cytocompatibility assay. The preparation of extracts was performed for 24 h. We did not pursue further incubation since that was not necessary based on the ISO 10993 guidelines. In accordance with the previous findings, no cytotoxicity was observed.

Others have used graphene oxide to cover the SS 316l to facilitate the attachment of SH-SY5Y cells [27]. In this study, the surface of the untreated SS 316l had a contact angle of 33.12°, which indicated high hydrophilicity. As mentioned, here, no signs of cytotoxicity were observed and cells grew and differentiated as usual. Further, in our study, the contact angle was 94.73°, which is significantly higher than the 33.12° which, in turn, is very low for stainless steel. The difference between the previous study and here is probably related to the fact that Tasnim et al. [27] used SS 316l grids and not a compact surface as we did here. This difference could likely account for this discrepancy in contact angle. Another point that supports the observation that microstructure affects contact angles comes from a study that fabricated electrodes for deep brain stimulation on the SS 316l surface using a UV nanosecond laser. In that study, the contact angle of SS 316l changed to almost 0° after patterning the electrodes. Namely, the newly generated grooved textured surface became superhydrophilic [28]. Further, studies on thin electrodes constructed by SS 316l are required to demonstrate their application in the construction of neuronal implants.

Despite the fact that the surface was characterized as hydrophobic [29], when the contact angle was greater than 90°, no changes in the cellular adhesion or proliferation were found. This may be related to the fact that superhydrophobic surfaces (>150°) are considered unfriendly for cells [30], and the 94.73° found here is very close to the limit between the hydrophilic/hydrophobic surface. Also, newer studies suggest that this limit (90°) has been arbitrarily set for hydrophilicity/hydrophobicity and other parameters have to be taken into consideration as well [31].

To this end, it should be mentioned that besides contact angle, there are other factors that affect cell adhesion and biomimicry such as surface energy, surface charge, surface roughness, presence of micropores, and stiffness [32]. Although we do not expect these parameters to significantly affect cellular adhesion in our case [since in SS 316l and TCP the number of cells attached at various times were the same (Figure 4)], these factors may account for the shorter neurite length observed in differentiated SH-SY5Y cells grown on SS 316l compared to TCP.

Previously, it was shown that the uncoated SS 316l surface is cytocompatible with platelets and smooth muscle cells but not with endothelial cells [33]. Our study expands the cytocompatibility repertoire of SS 316l.

## 5. Conclusions

We demonstrated that the SH-SY5Y cells easily adhered onto the surface of SS 316l and successfully differentiated into neuron-like cells upon RA treatment. The differentiation was apparent at the biochemical level, as shown by the increased expression of MAP2 at the morphological level through the neurite extension, albeit shorter than the ones generated on TCP. Further, SS 316l proved to be resistant to biocorrosion with only negligible amounts of metal wearing off the surface. In conclusion, our study suggests that uncoated SS 316l may be used in applications requiring the direct interaction of the material with neuronal cells, e.g., for the generation of neuronal stimulation electrodes and in other biomimetic applications. Further studies will be required on the potential applications of SS 316l as a neuronal implant and its exploitation in biomimetics overall.

## Figures and Tables

**Table 1:** Sequences of primers used in this study.

Name	Sequence (5′→3′)
*MAP2* Forward	TCTGCCTCCTTCTCCACCCC
*MAP2* Reverse	TCTGACTCCTTTTCCTTCTG
*NEFH* Forward	CTGGAGGCACTGAAAAGCA
*NEFH* Reverse	TCTTGACATTGAGCAGGTC
*HPRT-1* Forward	GCCCTGGCGTCGTGATTAGT
*HPRT-1* Reverse	AGCAAGACGTTCAGTCCTGTC

**Table 2:** XRF analysis for wear metals.

Metal	Concentration (μg/L)	Total Amount (μg)
Fe	36 ± 13	0.108 ± 0.039
Cr	16 ± 14	0.480 ± 0.420
Ni	22 ± 40	0.066 ± 0.012
Mn	105 ± 50	0.315 ± 0.015

## Data Availability

Data are contained within the article.
